# Association between the geriatric nutritional risk index and clinical outcomes among peritoneal dialysis patients: A meta-analysis

**DOI:** 10.1097/MD.0000000000038048

**Published:** 2024-05-03

**Authors:** Renjie Wang, Yuxiang Liang, Jiaojiao Jiang

**Affiliations:** a Department of Rehabilitation Medicine, West China Hospital, Sichuan University, Chengdu, People’s Republic of China.

**Keywords:** all-cause mortality, geriatric nutritional risk index, meta-analysis, peritoneal dialysis

## Abstract

**Background:**

To identify the relationship between the geriatric nutritional risk index (GNRI) and clinical outcomes in patients receiving peritoneal dialysis (PD).

**Methods:**

The PubMed, EBASE, Web of Science and CNKI databases were searched for available studies up to December 25, 2023. The primary outcome was all-cause mortality, and the secondary outcomes included the incidence of PD dropout, major adverse cardiac and cerebrovascular events (MACCEs), technique failure and peritonitis. Hazard ratios (HRs) and 95% confidence intervals (CIs) were combined to evaluate the predictive value of the GNRI for the occurrence of the above endpoints.

**Results:**

Ten cohort studies with 3897 patients were included. The pooled results demonstrated that a lower GNRI was significantly associated with a greater incidence of all-cause mortality (HR = 0.71, 95% CI: 0.55–0.91; *P* = .007). In addition, a decreased GNRI predicted the occurrence of dropout from PD (HR = 0.971, 95% CI: 0.945–0.998, *P* = .034) and MACCE (HR = 0.95, 95% CI: 0.92–0.98, *P* = .001). However, no significant associations of the GNRI with technique failure (*P* = .167) or peritonitis (*P* = .96) were observed.

**Conclusion:**

A low GNRI is significantly associated with poor clinical outcomes and might serve as a novel and valuable prognostic indicator among PD patients.

## 1. Introduction

Peritoneal dialysis (PD) is the most crucial renal replacement therapy for patients with end-stage renal disease (ESRD). The fundamental principle of PD lies in using the peritoneum as a biological semipermeable membrane to achieve transmembrane transport of substances, clear metabolic waste and excess fluids, and maintain water and electrolyte balance. Currently, there are more than 1000,000 PD patients globally, accounting for 10% to 15% of dialysis patients.^[1,2]^ It has been reported that up to 75% of patients with ESRD exhibit varying degrees of protein-energy wasting.^[3]^ Among patients undergoing maintenance PD, the substantial loss of protein in the dialysate increases the susceptibility to malnutrition compared to that in hemodialysis patients.^[4]^ Therefore, consideration of the nutritional status of PD patients is of utmost importance.

Malnutrition is closely related to poor prognosis among PD patients. In detail, PD patients with malnutrition may experience a greater risk of mortality and PD-related complications, such as major adverse cardiac and cerebrovascular events (MACCEs) and peritonitis.^[5]^ However, accurate assessment of the nutritional status of PD patients is currently a challenging issue.

The geriatric nutritional risk index (GNRI) was first reported by Bouillance et al in 2005^[6]^; it is based on the serum ALB concentration and body weight and is less influenced by subjective factors, making the assessment more objective and operationally simple. Previous studies have shown that the GNRI is a reliable indicator for assessing malnutrition in patients.^[7–9]^ Xiong et al demonstrated that a low GNRI was significantly related to an increased risk of mortality and cardiovascular events after including 19 cohort studies with 10,739 patients and indicated that the GNRI could serve as an effective tool to identify the risk for patients receiving hemodialysis.^[10]^ However, whether the GNRI could be applied as a novel and reliable indicator for predicting the clinical outcomes of PD patients remains unclear.

Therefore, the aim of this study was to further determine the relationship between the GNRI and clinical outcomes, including mortality and PD-related complications, in patients receiving PD based on the currently available evidence.

## 2. Materials and methods

This meta-analysis was conducted according to the Preferred Reporting Items for Systematic Review and Meta-Analyses 2020.^[11]^

### 2.1. Literature retrieval

In our study, the PubMed, EMBASE, Web of Science and CNKI databases were searched from inception to December 25, 2023. The following terms were used during the search: GNRI and PD. The specific search strategies used were as follows: GNRI AND PD. Moreover, MeSH terms and free texts were used, and the references of the included studies were reviewed.

### 2.2. Inclusion criteria

Studies that met the following criteria were included in our meta-analysis: patients who received PD for at least 1 month; the GNRI was calculated as follows: 14.89*serum albumin (g/dL) + 41.7*body weight/ideal body weight^[6]^; the association between the GNRI and clinical outcomes, including all-cause mortality, incidence of dropout of PD, MACCEs,^[12]^ technique failure and peritonitis,^[13]^ was explored, providing hazard ratios (HRs) with 95% confidence intervals (CIs) or similar effect sizes; and full texts were available.

### 2.3. Exclusion criteria

Studies that met the following criteria were excluded: were editorials, animal trials, case reports, reviews or letters; and had insufficient, overlapping or duplicated data.

### 2.4. Data collection

The following information was extracted from the included studies: the first author, publication year, sample size, country, disease, cutoff value of the GNRI, follow-up period, endpoint including all-cause mortality, incidence of PD dropout, MACCE, technique failure and peritonitis, and Newcastle–Ottawa Scale (NOS) and HR with 95% CI.

### 2.5. Methodological quality evaluation

The methodological quality was assessed using the NOS score, and studies with NOS scores >5 were defined as high-quality studies.^[14]^

In this meta-analysis, the literature search, selection, data collection and quality evaluation were also performed by 2 authors, and any disagreements were resolved by team discussion.

### 2.6. Statistical analysis

The analysis was performed with STATA version 15.0 software. The heterogeneity between studies was calculated by *I*^2^ statistics and the Q test. If significant heterogeneity was detected as *I*^2^ > 50% and/or *P* < .1, the random effects model was applied; otherwise, the fixed effects model was applied.^[15]^ The HR and 95% CI were combined to evaluate the relationship between the GNRI and clinical outcomes among PD patients.

## 3. Results

### 3.1. Literature selection process

**Figure 1.**
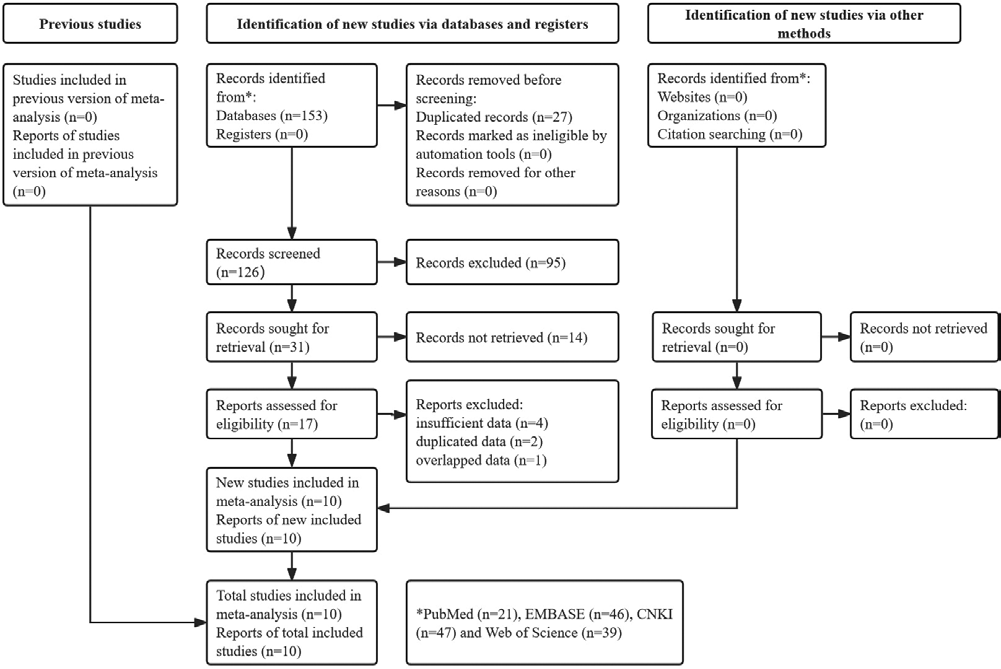
Prisma flow diagram of this meta-analysis.

One hundred and fifty-three records were found in the 4 databases, and 27 duplicated records were removed. After reviewing the titles and abstracts, 109 publications were removed. Finally, after reviewing the full texts, 10 studies were included in our meta-analysis.^[12,13,16–23]^ The detailed process is presented in Figure 1.

### 3.2. Basic characteristics of the included studies

A total of 3897 patients were enrolled, with sample sizes ranging from 45 to 1804. Most studies were from China or Japan and focused on patients with ESRD. The cutoff values of the GNRI ranged from 83.3 to 106.3. In addition, all studies were defined as high-quality studies with NOS scores ≥ 6. The specific information is shown in Table 1.

**Table 1:** Basic characteristics of included studies.

Author	Yr	Sample size	Country	Disease	Cutoff value of GNRI	Follow-up period	Endpoint	NOS
Kang^[16]^	2013	486	Korea	ESRD	89.6	1–169 mo		7
Lee^[12]^	2017	133	Korea	ESRD	94.0	51.1 mo	③	7
Li^[17]^	2019	447	China	ESRD	90.5	19.5 (9.8–42.6) mo; 20.4 (12.3–41.9) mo	②	8
Ren^[18]^	2019	1804	China	Mixed	94.55	39.3 (3–147.5) mo	①	7
Yang^[19]^	2020	252	China	ESRD	91.83	1.9 (0.8–3.1) yr	①③	6
Yang^[20]^	2021	276	China	ESRD	85.77	2.5 (1.4–4.0) yr	④	6
Uchiyama^[13]^	2022	116	Japan	Mixed	Continuous	46.2 (23.8–75.3) mo	④⑤	6
Li^[21]^	2023	118	China	Mixed	92.38	NR		6
Yabe^[22]^	2023	45	Japan	ESRD	83.3	32 (18–51) mo		6
Yildirim^[23]^	2023	220	Turkey	ESRD	106.3	33.5 (16–66.7) mo	①	7

①: all-cause mortality; ②: dropout of PD; ③: MACCE: major adverse cardiac and cerebrovascular event; ④: technique failure; ⑤: peritonitis.

ESRD = end-stage renal disease, GNRI = geriatric nutritional risk index, NOS = Newcastle-Ottawa Scale.

### 3.3. Associations between the GNRI score and all-cause mortality among PD patients

**Figure 2.**
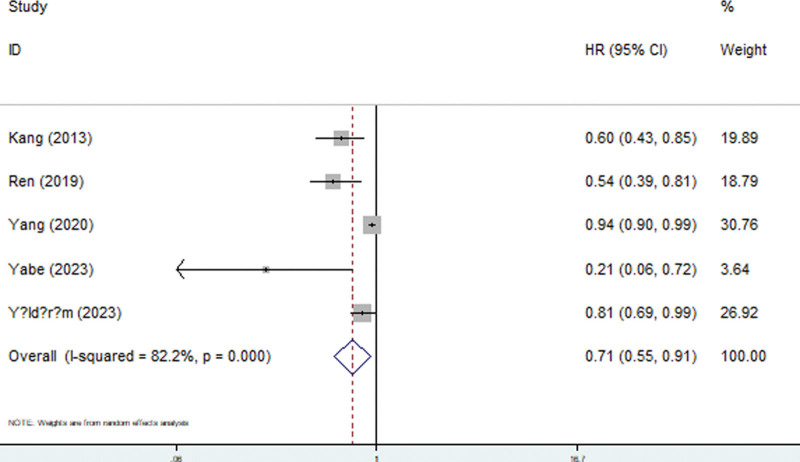
Forest plots for association between geriatric nutritional risk index and all-cause mortality among peritoneal dialysis patients.

Five included studies explored the relationship between the GNRI and all-cause mortality in PD patients.^[16,18,19,22,23]^ The pooled results demonstrated that the GNRI was significantly associated with all-cause mortality and that PD patients with a lower GNRI experienced a greater risk of all-cause mortality (HR = 0.71, 95% CI: 0.55–0.91, *P* = .007; *I*^2^ = 82.2%, *P* < .001) (Fig. 2) (Table 2).

**Table 2:** Results of meta-analysis.

	No. of studies	Hazard ratio	95% confidence interval	*P* value	*I * ^2^	*P* value
All-cause mortality	5	0.71	0.55–0.91	.007	82.2%	<.001
Dropout of PD	1	0.971	0.945–0.998	.034	-	-
Major adverse cardiac and cerebrovascular event	3	0.95	0.92–0.98	.001	40.1	.188
Technique failure	2	0.98	0.96–1.01	.167	0.0	.853
Peritonitis	1	0.877	0.68–1.136	.96	-	-

PD = peritoneal dialysis.

### 3.4. Associations between the GNRI and other outcomes among PD patients

**Figure 3.**
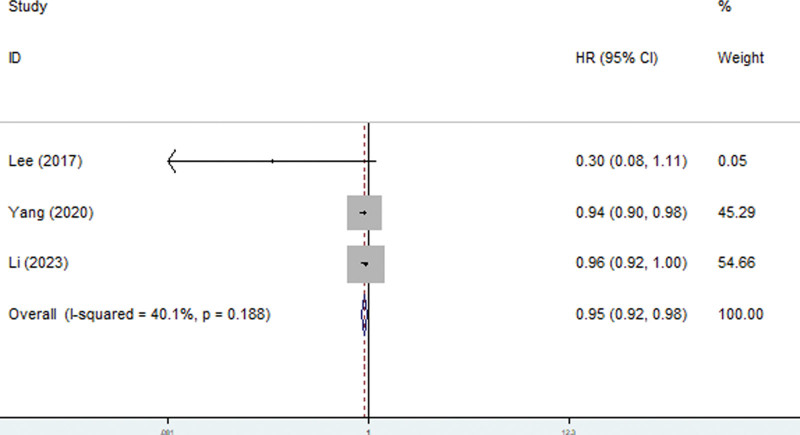
Forest plots for association between geriatric nutritional risk index and major adverse cardiac and cerebrovascular events among peritoneal dialysis patients.

Three studies explored the relationship between the GNRI and MACCEs.^[12,19,21]^ The pooled results showed that a lower GNRI predicted a greater incidence of MACCEs (HR = 0.95, 95% CI: 0.92–0.98, *P* = .001; *I*^2^ = 40.1%, *P* = .188) (Fig. 3). In addition, Li et al reported that the GNRI was also significantly related to PD dropout (HR = 0.971, 95% CI: 0.945–0.998, *P* = .034).^[17]^

However, 2 studies identified an association between the GNRI and technique failure, and the pooled results indicated that no significant relationship between the GNRI and the occurrence of technique failure was observed (HR = 0.98, 95% 0.96–1.01; *P* = .167).^[13,20]^ Furthermore, according to the data of Uchiyama et al, the GNRI was not associated with the incidence of peritonitis among PD patients (HR = 0.877, 95% CI = 0.68–1.136, *P* = .96)^[13]^ (Table 2).

## 4. Discussion

Our meta-analysis demonstrated that the GNRI was significantly associated with all-cause mortality, MACCEs and PD dropout among PD patients. Overall, a low GNRI might serve as a novel and valuable risk factor for poor clinical outcomes in PD patients based on the currently available evidence.

According to our results, malnutrition, represented by a low GNRI, clearly predicted an increased risk of mortality. This finding may be related to multiple factors. First, malnutrition can compromise the immune system, increasing patient susceptibility to infections. In PD patients, especially those with compromised immune systems, this vulnerability may heighten the risk of mortality.^[24,25]^ Second, malnutrition can impede wound healing and postoperative recovery, including the rehabilitation process after peritoneal catheter insertion. Wound healing failure may lead to infections and other complications, increasing the risk of mortality.^[26]^ Third, PD patients already have a greater risk of cardiovascular diseases, and poor nutritional status may further exacerbate this risk. Fourth, malnutrition can lead to electrolyte imbalances, a common issue in PD patients, potentially causing abnormalities in heart, nerve, and muscle functions and increasing the risk of mortality.^[27,28]^ In addition, malnutrition may increase the risk of decreased bone density and fractures. In PD patients, metabolic issues related to bones could be severe complications associated with an increased mortality rate.^[29]^ Furthermore, malnutrition can induce a systemic chronic inflammatory response, negatively affecting various organ systems and further increasing the risk of mortality.^[30,31]^ Overall, PD patients are already at high risk, and malnutrition may exacerbate existing health issues, leading to severe complications and an increased risk of mortality. Therefore, maintaining good nutritional status in PD patients is crucial for improving survival rates and preventing complications.

Malnutrition is also closely associated with an increased risk of MACCEs. Malnutrition may lead to electrolyte imbalance, particularly related to sodium and potassium, thereby increasing the risk of hypertension. Hypertension is a major risk factor for cardiovascular diseases and is closely associated with MACCEs. In addition, as mentioned above, malnutrition may induce a systemic chronic inflammatory response, triggering the release of inflammatory mediators, increasing the likelihood of endothelial damage, and promoting the development of atherosclerosis.^[32,33]^ Changes in myocardial load due to malnutrition can cause abnormalities in cardiac structure and function, potentially increasing the risk of cardiovascular events. Malnutrition may be associated with insulin resistance and abnormal glucose metabolism, increasing the risk of diabetes, an independent risk factor for cardiovascular diseases.^[34,35]^ Electrolyte imbalances, such as abnormalities in potassium, induced by malnutrition can impact cardiac electrical activity, increasing the risk of arrhythmias and cardiac events. Malnutrition may affect the normal function of the vascular endothelium, leading to abnormal regulation of vasodilation and constriction, thereby increasing the risk of cardiovascular events.^[36,37]^

Notably, although no significant association of the GNRI with technique failure or peritonitis was observed, only 2 included studies performed relevant explorations. Therefore, it is still necessary to identify the predictive role of the GNRI for technique failure and peritonitis among PD patients in future research.

There are several limitations that should be noted. First, the overall sample size was relatively small, and all included studies were from Asian countries, which may cause bias. Second, the associations between the GNRI and other clinical outcomes, such as cardiovascular mortality and infection, could not be explored due to the lack of relevant data. Third, the cutoff values of the GNRI varied among the included studies, and we were unable to further determine the optimal critical values of the GNRI for predicting the clinical outcomes of PD patients in our meta-analysis.

## 5. Conclusion

A low GNRI is significantly associated with poor clinical outcomes and might serve as a novel and valuable prognostic indicator among PD patients. However, more high-quality studies are still needed to further verify the above findings.

## Author contributions

**Conceptualization:** Renjie Wang, Jiaojiao Jiang.

**Data curation:** Yuxiang Liang.

**Formal analysis:** Renjie Wang.

**Methodology:** Renjie Wang, Yuxiang Liang.

**Software:** Yuxiang Liang.

**Supervision:** Jiaojiao Jiang.

**Writing – original draft:** Renjie Wang.

**Writing – review & editing:** Jiaojiao Jiang.
